# Case Report: Lessons Learned From Subsequent Autologous and Allogeneic Hematopoietic Stem Cell Transplantations in a Pediatric Patient With Relapsing Polychondritis

**DOI:** 10.3389/fimmu.2022.812927

**Published:** 2022-03-10

**Authors:** Saskia R. Veldkamp, Marc H. A. Jansen, Joost F. Swart, Caroline A. Lindemans

**Affiliations:** ^1^ Center for Translational Immunology, Wilhelmina Children’s Hospital, University Medical Center Utrecht, Utrecht, Netherlands; ^2^ Pediatric Rheumatology and Immunology, Wilhelmina Children’s Hospital, University Medical Center Utrecht, Utrecht, Netherlands; ^3^ Blood and Bone Marrow Transplantation, Princess Máxima Center for Pediatric Oncology, Utrecht, Netherlands

**Keywords:** case report, relapsing polychondritis, autologous hematopoietic stem cell transplantation, allogeneic hematopoietic cell transplantation, autoimmune disease, cytotoxic T cells

## Abstract

**Background:**

Autologous hematopoietic stem cell transplantation (autoHSCT) is increasingly being recognized as a treatment option for severe refractory autoimmune diseases (AD). However, efficacy is hampered by high relapse rates. In contrast, allogeneic HSCT (alloHSCT) has high potential to cure AD, but is associated with significant morbidity and mortality, and data in AD are limited. Experience with autoHSCT in relapsing polychondritis, a rare episodic inflammatory disorder characterized by destruction of cartilage, is scarce and alloHSCT has not been described before.

**Case Presentation:**

Here, we present a case of a 9-year-old girl who was diagnosed with relapsing polychondritis, with severe airway involvement requiring a tracheostomy. The disease proved to be steroid-dependent and refractory to a wide array of disease-modifying anti-rheumatic drugs and biologicals. After an autoHSCT procedure, the disease became inactive for a short period of time, until the patient experienced a relapse after 31 days, accompanied by repopulation of effector/memory CD8^+^ T cells. Because of persistent inflammation and serious steroid toxicity, including severe osteoporosis, growth restriction, and excessive weight gain, the patient was offered an alloHSCT. She experienced transient antibody-mediated immune events post-alloHSCT, which subsided after rituximab. She ultimately developed a balanced immune reconstitution and is currently still in long-term disease remission, 8 years after alloHSCT.

**Conclusion:**

This case adds to the few existing reports on autoHSCT in relapsing polychondritis and gives new insights in its pathogenesis, with a possible role for CD8^+^ T cells. Moreover, it is the first report of successful alloHSCT as a treatment for children with this severe autoimmune disease.

## Introduction

In the past 25 years, autologous hematopoietic stem cell transplantation (autoHSCT) has been used to treat severe refractory autoimmune diseases (AD) in adults and children (1, 2). The aim of autoHSCT is to reset the immune system by eliminating autoreactive T and B cells with high-dose immunosuppression and promoting the generation and outgrowth of an immune system with a new self-tolerant immune repertoire. An increasing amount of evidence supports autoHSCT in a wide range of AD, including multiple sclerosis (MS), systemic sclerosis (SSc), and Crohn’s disease (3–6). While some patients achieve long-term remission, others experience reactivation of their disease post-autoHSCT (7). In contrast, allogeneic HSCT (alloHSCT) has a higher curative potential, but is associated with significant morbidity and mortality, including graft-versus-host-disease (GvHD) and viral reactivations. Experience with alloHSCT in refractory AD is therefore limited and mainly restricted to pediatric practice, with immune cytopenias as the predominant indication (8, 9). Here, we report a case of a girl with severe steroid-dependent relapsing polychondritis, a rare inflammatory disorder characterized by recurrent episodes of inflammation and deterioration of cartilaginous structures. This patient’s disease was refractory to azathioprine, methotrexate, infliximab, cyclophosphamide and anakinra, and relapsed one month after autoHSCT. This relapse was concurrent with the repopulation of effector/memory CD8^+^ T cells. After unsuccessful treatment attempts with tacrolimus, tocilizumab and abatacept, long-term remission was eventually induced by alloHSCT. This unique case adds to the scarcely available literature on autoHSCT in relapsing polychondritis, provides insights in the pathogenesis of this disease, and is the first report of successful alloHSCT as a rescue treatment for children with this severe autoimmune disorder.

## Case Description

An 8-year-old girl was admitted to the Intensive Care Unit (ICU) twice in October 2010 with acute respiratory distress due to an upper airway obstruction. At laryngoscopy, a subglottic stenosis was seen and blood results showed an iron deficiency anemia. In the preceding months, she had experienced weight loss and fever, with no response to antibiotic treatment. Granulomatosis with Polyangiitis was initially considered as diagnosis, but anti-neutrophil cytoplasmic antibodies (ANCA) test results were negative. Methylprednisolone pulse therapy was administered during the second admission with marked improvement of the patient’s condition, and she was discharged home with oral steroids and azathioprine. However, during steroid tapering the girl again developed an inspiratory stridor, as well as a saddle nose and pain complaints at the costochondral junctions. She was diagnosed with relapsing polychondritis at the end of December 2010, upon which the steroid dosage was increased, azathioprine was switched to methotrexate (MTX) and infliximab was started. Nevertheless, the patient was readmitted to the ICU shortly thereafter because of acute respiratory distress requiring intubation, and a tracheostomy was performed. Moreover, she developed arthritis of the temporomandibular joint, fever, and increased costochondral pain, with rising C-reactive protein (CRP) and erythrocyte sedimentation rate (ESR) levels. Methylprednisolone pulse therapy ameliorated symptoms and lowered inflammation markers, but exacerbations were still frequent. Consequently, intravenous cyclophosphamide was started, and infliximab was withdrawn. In the following 6 months, she received monthly doses of 750mg/m^2^ cyclophosphamide. Although no exacerbations occurred, disease remission was not achieved as she had persistent complaints of pain in the chest, jaws and limbs, accompanied by elevated CRP levels (61 - 111 mg/L). Anakinra was added to the regimen of MTX and steroids in July 2011, because of a few successful case reports, but had no effect. An F-18-FDG positron emission tomography (PET) scan confirmed persistent disease activity in the cartilage of the larynx, bronchial tree and ribs. Because of this persistent inflammation and the inability to taper the steroids below 1 mg/kg/d, causing side effects such as steroid-induced Cushing syndrome, the patient was referred to our center for an autoHSCT, with the aim of resetting the immune system and restoring self-tolerance.

### AutoHSCT

**Figure 1:**
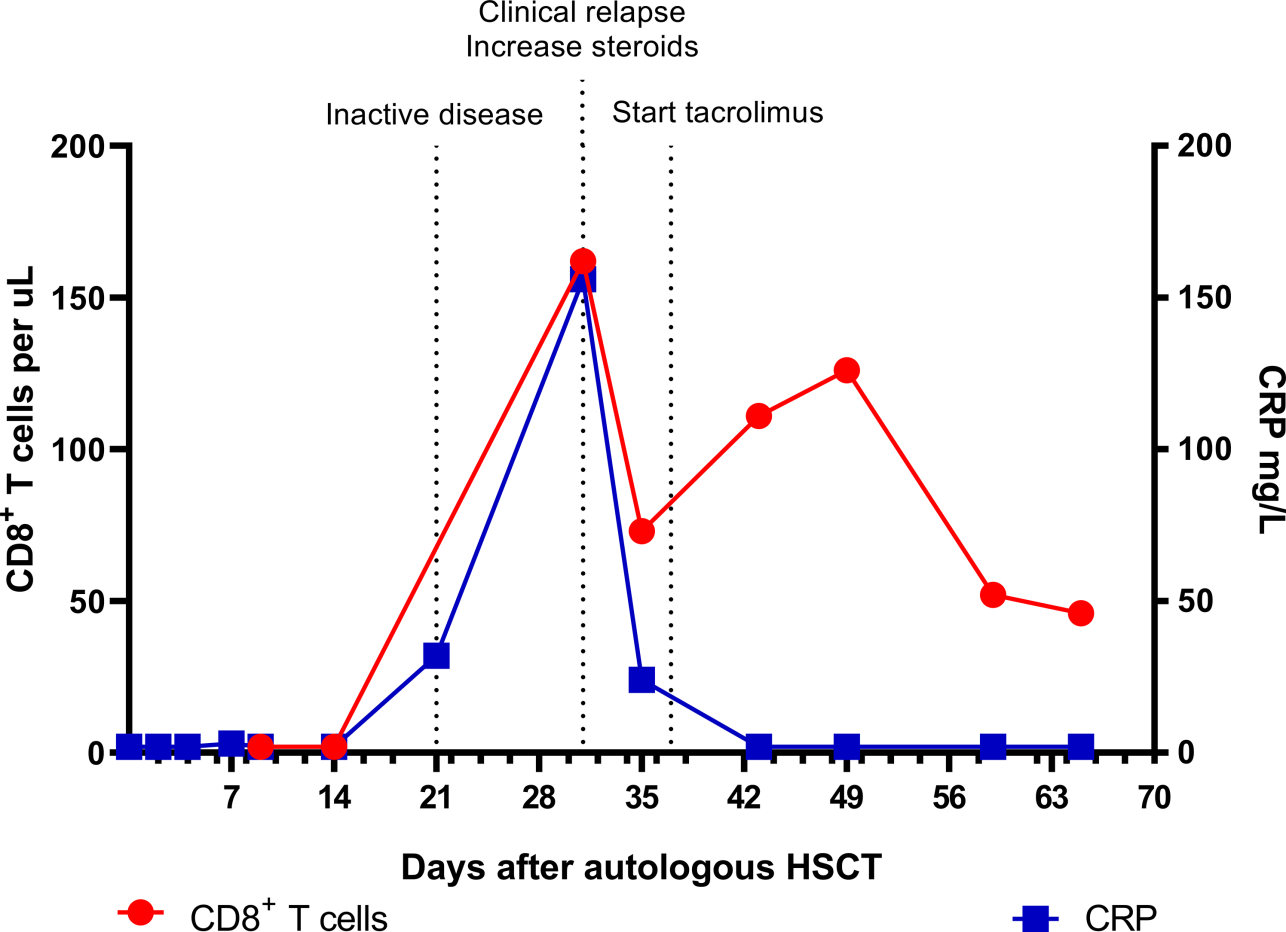
Absolute CD8^+^ T cell numbers and CRP levels in the early phase after autologous HSCT. Inactive disease status on day 21 was based on a normal F-18-FDG PET scan and absence of symptoms. CRP, C-reactive protein; HSCT, hematopoietic stem cell transplantation; PET, positron emission tomography.

In November 2011, the patient received an autoHSCT (5.85 x 10^6^ CD34^+^ cells/kg body weight) after stem cell mobilization with cyclophosphamide and recombinant granulocyte colony stimulating factor (G-CSF) and a conditioning regimen consisting of antithymocyte globulins (ATG) 10 mg/kg in 4 days (-10, -10, -9, -7), cyclophosphamide 120 mg/kg in 2 days (-7, -6) and fludarabine 150 mg/m^2^ in 4 days (-5, -4, -3, -2). After the autoHSCT, which was uncomplicated, all symptoms disappeared, CRP level normalized, and an F-18-FDG PET scan 3 weeks post-HSCT scan showed complete remission. Oral steroids were gradually tapered to 0.3 mg/kg/d. However, 31 days after autoHSCT the patient experienced a relapse of costochondral pain and CRP level increased again to 156 mg/L. Flow cytometric immunophenotyping showed repopulation of specifically CD8-positive(^+^) T cells (**Figure 1**). Of all CD8^+^ T cells, 91.3% were of the effector/memory type (CD27^+^CD45RA^-^/CD27^-^CD45RA^-^/CD27^-^CD45RA^+^) and 8.7% were naïve (CD27^+^CD45RA^+^). Almost all (97.2%) had an activated phenotype (CD38^+^HLA-DR^+^). A viral infection was considered as a potential trigger for this repopulation, but analysis of the CD3^+^ T cell Receptor (TCR)-Vbeta repertoire showed a polyclonal pattern and quantitative PCR measurements for Epstein-Barr virus (EBV), cytomegalovirus (CMV), Human Herpesvirus 6 (HHV6), and adenovirus were negative.

**Figure 2:**
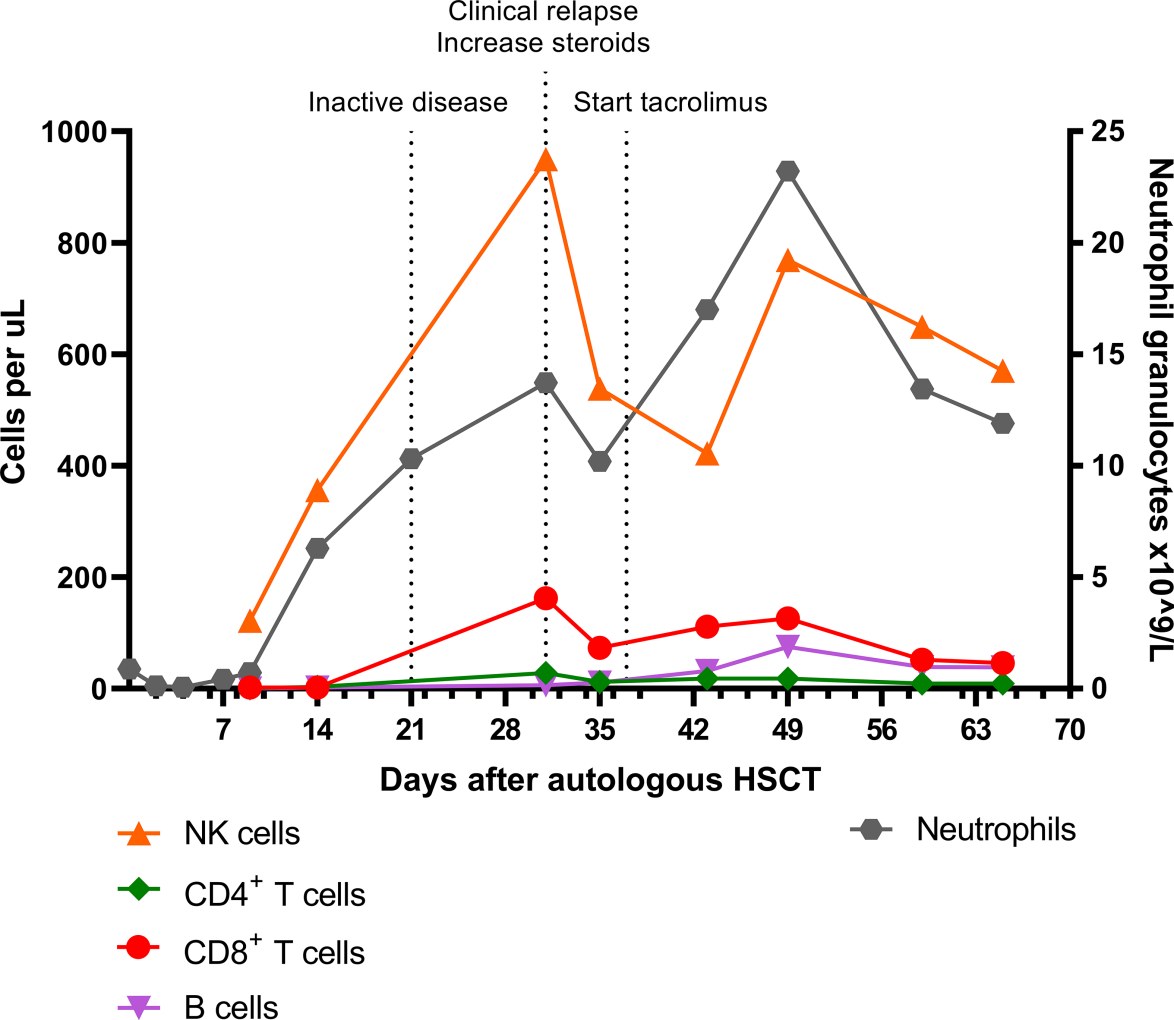
Absolute NK cell, CD4^+^ T cell, CD8^+^ T cell, B cell, and neutrophil granulocyte numbers in the early phase after autologous HSCT. Inactive disease status on day 21 was based on a normal F-18-FDG PET scan and absence of symptoms. NK cell, Natural Killer cell; HSCT, hematopoietic stem cell transplantation; PET, positron emission tomography.

**Figure 3:**
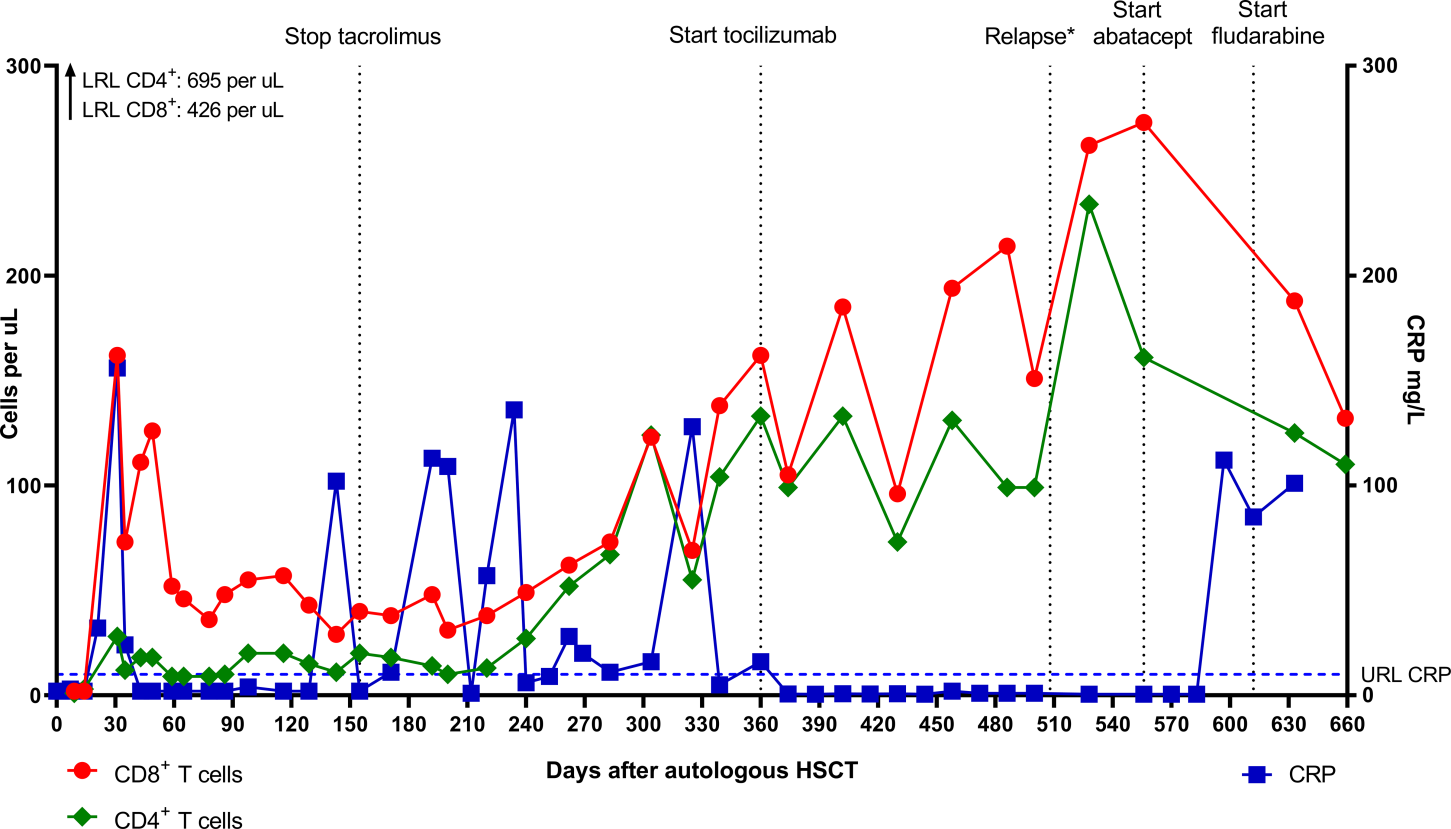
Absolute CD8^+^ and CD4^+^ T cell numbers and CRP levels after autologous HSCT. *Relapse on day 508 was characterized by clinical symptoms and disease activity on F-18-FDG PET scan. CRP level was probably not elevated due to the masking effect of tocilizumab (10). LRL, lower reference limit; URL, upper reference limit; CRP, C-reactive protein; HSCT, hematopoietic stem cell transplantation; PET, positron emission tomography.

In contrast to the CD8^+^ T cells, the CD4^+^ T cell and B cell (CD19^+^) counts remained low. Absolute numbers of Natural Killer (NK) cells (CD3^-^CD16^+^56^+^) and neutrophils started rising early after autoHSCT (**Figure 2**). Steroid dosage was increased to 1.3 mg/kg/d, after which CD8^+^ T cell, NK cell and CRP levels rapidly declined. In addition, tacrolimus was started as a steroid-sparing agent, with the aim of slowing down CD8^+^ T cell repopulation so that new thymic output could arise and induce self-tolerance. In the following year, steroid dosage could be reduced to 0.3 mg/kg/d, but several attempts at further tapering resulted in disease flares, as illustrated by the CRP elevations in **Figure 3**. Tacrolimus had to be withdrawn due to renal toxicity and the patient suffered from serious steroid-induced side effects, including severe osteoporosis, growth restriction, hypertension and Cushingoid features with excessive weight gain. After approximately 300 days post-HSCT, new thymic output appeared as reflected by rising CD8^+^ and CD4^+^ T cell numbers with a 48% and 44% naïve phenotype, respectively. However, also in this new situation further tapering of steroids resulted in a disease flare (day 325, **Figure 3**). This confirmed that autoHSCT had failed to restore self-tolerance. In search of a novel treatment strategy, tocilizumab was started in November 2012, which led to clinical improvement and made further steroid tapering possible (0.08 mg/kg every other day), until the patient relapsed again in April 2013. This relapse was characterized by a return of clinical symptoms (costochrondral pain, loss of appetite) and increased activity on an F-18-FDG PET scan. CRP level was probably not elevated at this time due to the masking effect of tocilizumab (10). After an unsuccessful attempt with abatacept, we concluded that for this patient with steroid-dependent relapsing polychondritis with severe steroid toxicity there were no other treatment options left, except for alloHSCT. During the screening process for alloHSCT, fludarabine was given in order to be able to decrease the steroid dosage.

### AlloHSCT

**Figure 4:**
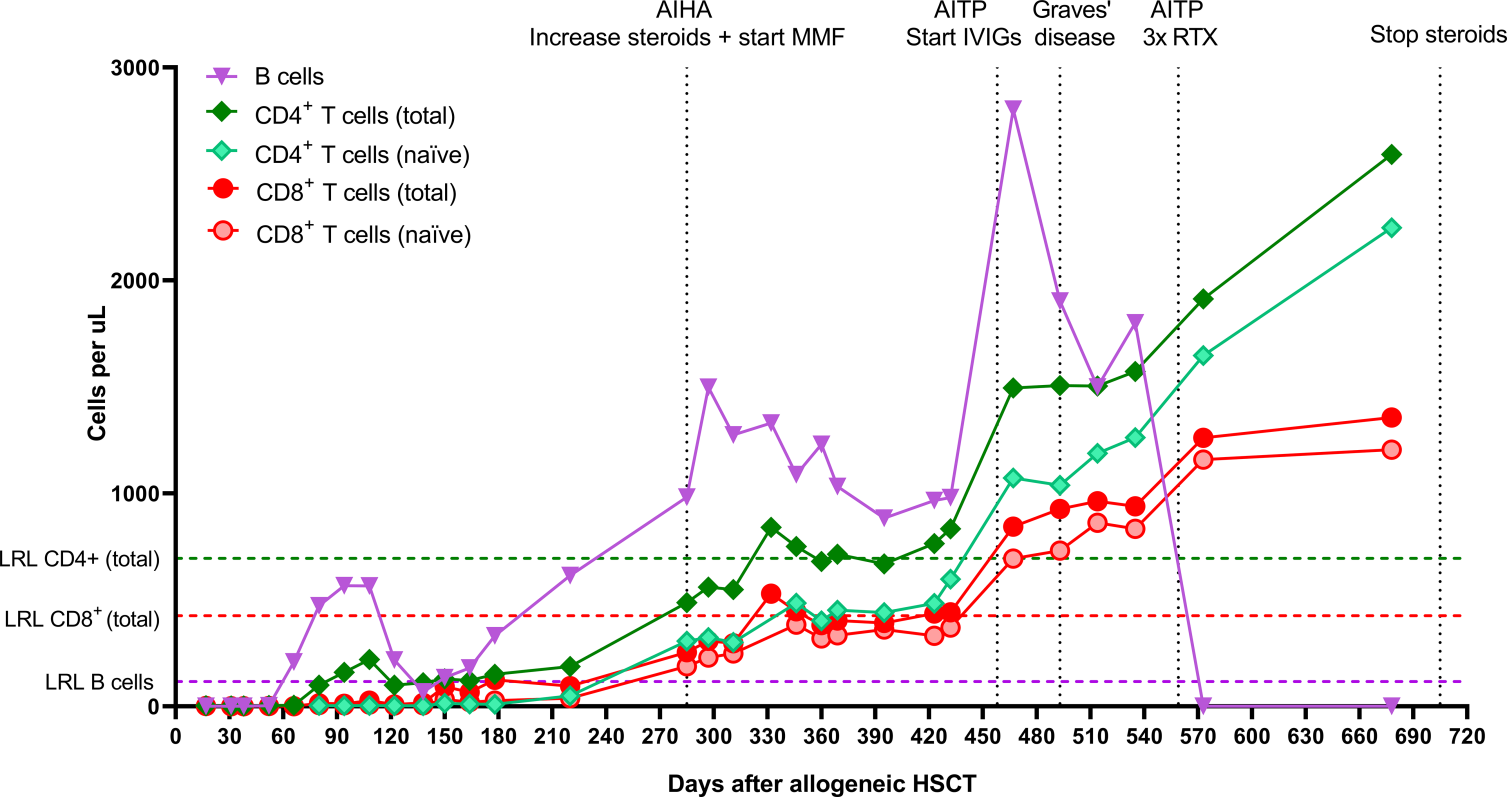
Absolute CD8^+^ T cell, CD4^+^ T cell, and B cell numbers after allogeneic HSCT. CD27^+^CD45RA^+^ T cells were characterized as naïve T cells. AIHA, autoimmune hemolytic anemia; MMF, mycophenolate mofetil; AITP, autoimmune thrombocytopenia; IVIGs, intravenous immunoglobulins; RTX, rituximab; LRL, lower reference limit; HSCT, hematopoietic stem cell transplantation.

Almost two years post-autoHSCT, after patient and parents both agreed, the now 11-year old patient received a cord blood transplant (5/6 HLA match, 0.12 x 10^6^ CD34^+^ cells/kg body weight) after reduced intensity conditioning with busulfan at a cumulative AUC of 60 in 3 days (-5, -4, -3), fludarabine 160 mg/m2 in 4 days (-5, -4, -3, -2), and early alemtuzumab as serotherapy. Alemtuzumab (1 mg/kg in 3 days) was chosen because of the prior ATG during the conditioning for autoHSCT (risk of anti-ATG-antibody formation) and for maximal recipient lymphodepletion. It was given very early (day -21 – day -19) to not impact immune recovery post-transplant. GvHD prophylaxis consisted of prednisolone, ciclosporin and mycophenolate mofetil (MMF). The early post-alloHSCT course was uncomplicated with remission of inflammation and symptoms, no serious toxicity, early and stable engraftment, and full donor chimerism. The earlier established benchmark of a CD4 count of >50 x 10^6^/L within 100 days, correlating with survival in pediatric alloHSCT, was easily reached (11, 12). New thymic output, as reflected by rising naïve CD4^+^ and CD8^+^ T cell counts, was observed 285 days post-alloHSCT (**Figure 4**). Both T cell (total) counts reached normal reference limits 332 days post-alloHSCT. The patient developed several immune-mediated events during immune reconstitution, including Graves’ disease and donor-induced autoimmune hemolytic anemia and thrombocytopenia, which were treated with steroids, MMF, and intravenous immunoglobulins (IVIGs), and only subsided after a rituximab course (375 mg/m2 weekly, 3x). These events were considered unrelated to her prior auto-immune disease and were of temporary nature. Six years post-alloHSCT, the last immunosuppressive medication (MMF) was withdrawn and recently, the tracheostomy tube was removed and the trachea was reconstructed successfully. She has received hormone replacement therapy because of secondary growth restriction and hypergonadotropic hypogonadism. The patient is currently still in long-term disease remission, 8 years after alloHSCT.

## Discussion

Relapsing polychondritis is a rare episodic inflammatory disorder involving immune-mediated destruction of cartilaginous structures. Disease onset is most likely between the ages of 40 and 60, but the disease has been described in children as well (13, 14). Laryngeal chondritis occurs in more than half of patients, and chronic laryngotracheobronchial involvement represents advanced disease with poor prognosis and increased mortality risk. Treatment has not been standardized and, although symptom control can be achieved, it usually does not prevent disease progression (15, 16). Only three (adult) cases that received autoHSCT have been published, two of which reported complete remission after 18 and 21 months follow-up (17, 18). The third case was reported in a retrospective study on rituximab in relapsing polychondritis and had previously received autoHSCT, indicating that no remission was achieved (19). No reports on alloHSCT in relapsing polychondritis currently exist.

Little is known about the pathogenesis of relapsing polychondritis, although there is some evidence of cell-mediated autoimmunity playing a role (20). Besides the strong association with the genetic allele HLA-DR4, T cells have been found in affected cartilage, and T cell responses specific to collagen type II peptides were reported (21–24). Also, decreased regulatory T cell (Treg) counts and a less diverse TCR repertoire were observed in patients with relapsing polychondritis compared to healthy controls (25, 26). However, the presence of autoantibodies directed against collagens and other cartilage matrix components suggests that humoral autoimmunity is involved as well (27, 28). This case report provides further insights in the pathogenesis, as the relapse after autoHSCT was concurrent with an increase in CD8^+^ T cells, suggesting that this cytotoxic cell type plays a role in relapsing polychondritis.

The rationale behind autoHSCT in AD is that after depletion of autoreactive T and B cells, a reset and naïve immune system can be regenerated from the stem cell graft, although the exact mechanisms by which self-tolerance is induced are still unknown. It is thought that successful induction of disease remission after autoHSCT relies primarily on the complete renewal of the CD4^+^ TCR repertoire through thymopoeisis, a process that can require at least 6 months and sometimes years, with a particularly important role for Tregs (1, 29–31). Reconstitution of CD8^+^ T cells occurs earlier, within 1 to 6 months post-autoHSCT, due to proliferation of either cells contained within the graft or residual cells that escaped the pre-transplant conditioning therapy (32–40). Conserved T cell clones have been demonstrated after autoHSCT in both SSc and MS patients (36, 38). In patients with MS, the expansion of pre-existing CD8^+^ T cell clones was not associated with a different clinical outcome, suggesting that these clones were either not autoreactive, or no longer able to induce disease activity post-autoHSCT (36, 39, 40). In our patient, however, the increase in CD8^+^ T cells on day 31 post-transplantation was accompanied by rising CRP levels and a relapse in symptoms, strongly suggesting that these were expanding autoreactive T cells that had not been eliminated by the lymphodepleting conditioning regimen and were able to induce disease reactivation. Whether this can be contributed to insufficient lymphodepletion is unclear, as cases with persisting or re-emerging autoreactive immune cells without concomitant clinical relapse have also been described, indicating that other mechanisms besides lymphodepletion are involved in obtaining disease control after autoHSCT (41–44). Indeed, “reprogramming” of residual autoreactive cells towards a more anti-inflammatory/regulatory phenotype and the restoration of regulatory networks have been proposed to also take part in the process of restoring self-tolerance (44–48). We may thus speculate that the expanding CD8^+^ T cells in our patient not only escaped the lymphodepleting regimen, but also escaped the immunomodulatory effects of the autoHSCT, resulting in clinical relapse. A viral infection triggering the rise in CD8^+^ T cells cannot be ruled out, even though the patient did not show any related symptoms and the most common post-HSCT viral reactivations were tested negative by quantitative PCR.

In the two years post-autoHSCT, complete T cell reconstitution was never observed, most likely due to extensive steroid treatment (49–52). After alloHSCT, however, full thymopoietic recovery and T cell reconstitution did occur, despite the extensive immunosuppression and two transplants. While alemtuzumab can cause unintentional lymphodepletion of the graft due to its prolonged half-life, it is highly unlikely that this occurred in our patient, since we chose to administer alemtuzumab very early pre-transplant for this reason and lymphocyte recovery post-transplant was not delayed (53). The development of a more balanced immune reconstitution over time may have contributed to the subsiding of the secondary immune-mediated diseases post-alloHSCT.

Although a significant fraction of patients with AD relapse after autoHSCT, many do become responsive to conventional treatment again (54). Our patient, too, required less steroids for symptom control after transplantation compared to before. Nevertheless, further tapering proved impossible and in the end she had to undergo alloHSCT due to persistent inflammation and severe steroid toxicity. AlloHSCT in AD has curative potential as it replaces a patient’s dysfunctional immune system with an allograft from a healthy donor. Whereas autoHSCT is increasingly being adopted as a treatment option in severe AD, there is limited experience with alloHSCT in these patients, and data are scattered (2, 55). Both long-term complete remissions and relapses have been reported after alloHSCT (2, 56–58). Because alloHSCT is associated with significant morbidity and mortality, including GvHD, it is currently only recommended for patients with refractory, life-threatening autoimmune disease. Data from the EBMT registry revealed that of the 128 patients who received alloHSCT for refractory autoimmune disease between 1997 and 2014, 20.8% had developed grade II-IV acute GvHD at 100 days post-transplant, and 27.8% had developed chronic GvHD at 5 years. Transplant-related mortality (TRM) and relapse rate were both 20% at 5 years (8). In contrast, the 5-year TRM in the 1,951 patients who underwent autoHSCT between 1994 and 2015 was 5% and the relapse rate was 46% (9). Thus, autoHSCT appears safer than alloHSCT, but has a higher risk of relapse. However, when comparing these two types of transplantation, it is important to note the recent advances that have been made in the field of alloHSCT. Novel conditioning approaches using reduced intensity regimens and individualized dosing strategies have been shown to optimize lymphodepletion and influence TRM, and improved diagnostics, treatment and prophylaxis have reduced infectious complications post-alloHSCT (8, 11, 12, 59–61). Multivariate analysis with EBMT registry data identified age <18 years and more recent year of transplant to be significantly associated with improved progression-free survival and lower TRM in patients with refractory AD (8). Thus, alloHSCT is becoming safer and may become a sensible option for patients with severe refractory AD, children in particular. In autoHSCT, too, younger age was associated with lower TRM and improved survival, stressing the importance of timely consideration of HSCT (9).

In any case where HSCT is considered – autologous or allogeneic – for the treatment of AD, a thorough evaluation is required by an experienced transplantation team to weigh the risks of the transplantation against the burden of the disease. Careful selection and screening of patients, including evaluation of cardiopulmonary fitness and viral serological status, are vital in this process, as well as involving the patient and family early in the decision (2, 62).

## Conclusion

The case we presented here provides unique evidence of HSCT in relapsing polychondritis, as well as new insights in its pathogenesis, suggesting a possible role for CD8^+^ T cells. Moreover, it supports alloHSCT as a sensible option in the treatment of severe relapsing polychondritis, especially in pediatric cases with serious steroid toxicity and proven failure of autoHSCT. However, as clearly not every patient with multi-refractory autoimmune disease is able to deal with the toxicity of two sequential stem cell transplantations, physicians will have to carefully weigh the risks and chances of autoHSCT versus alloHSCT for patients with severe AD.

## Data Availability Statement

The original contributions presented in the study are included in the article/supplementary material. Further inquiries can be directed to the corresponding author.

## Ethics Statement

Written informed consent was obtained from the individual(s) for the publication of any potentially identifiable images or data included in this article.

## Author Contributions

SV wrote the case report. MJ, JS, and CL critically reviewed and revised the report. JS and CL were also involved as treating physicians in this case. All authors issued final approval for the final version to be submitted.

## Conflict of Interest

The authors declare that the research was conducted in the absence of any commercial or financial relationships that could be construed as a potential conflict of interest.

## Publisher’s Note

All claims expressed in this article are solely those of the authors and do not necessarily represent those of their affiliated organizations, or those of the publisher, the editors and the reviewers. Any product that may be evaluated in this article, or claim that may be made by its manufacturer, is not guaranteed or endorsed by the publisher.

## References

[1] Swart JF Delemarre EM van Wijk F Boelens J-J Kuball J van Laar JM. Haematopoietic Stem Cell Transplantation for Autoimmune Diseases. Nat Rev Rheumatol (2017) 13(4):244–56. doi: 10.1038/nrrheum.2017.7 28228650

[2] Abinun M Slatter MA. Haematopoietic Stem Cell Transplantation in Paediatric Rheumatic Disease. Curr Opin Rheumatol (2021) 33(5):387–97. doi: 10.1097/BOR.0000000000000823 34261117

[3] Alexander T Greco R Snowden JA. Hematopoietic Stem Cell Transplantation for Autoimmune Disease. Annu Rev Med (2021) 72(1):215–28. doi: 10.1146/annurev-med-070119-115617 33106103

[4] Atkins HL Bowman M Allan D Anstee G Arnold DL Bar-Or A. Immunoablation and Autologous Haemopoietic Stem-Cell Transplantation for Aggressive Multiple Sclerosis: A Multicentre Single-Group Phase 2 Trial. Lancet (2016) 388(10044):576–85. doi: 10.1016/S0140-6736(16)30169-6 27291994

[5] van Laar JM Farge D Sont JK Naraghi K Marjanovic Z Larghero J. Autologous Hematopoietic Stem Cell Transplantation vs Intravenous Pulse Cyclophosphamide in Diffuse Cutaneous Systemic Sclerosis: A Randomized Clinical Trial. JAMA (2014) 311(24):2490–8. doi: 10.1001/jama.2014.6368 25058083

[6] Lindsay JO Allez M Clark M Labopin M Ricart E Rogler G. Autologous Stem-Cell Transplantation in Treatment-Refractory Crohn’s Disease: An Analysis of Pooled Data From the ASTIC Trial. Lancet Gastroenterol Hepatol (2017) 2(6):399–406. doi: 10.1016/S2468-1253(17)30056-0 28497755

[7] Dominique F Myriam L Alan T Athanasios F Gian Luigi M Jaap Van L. Autologous Hematopoietic Stem Cell Transplantation for Autoimmune Diseases: An Observational Study on 12 Years’ Experience From the European Group for Blood and Marrow Transplantation Working Party on Autoimmune Diseases. Haematologica (2010) 95(2):284–92. doi: 10.3324/haematol.2009.013458 PMC2817032 19773265

[8] Greco R Labopin M Badoglio M Veys P Furtado Silva JM Abinun M. Allogeneic HSCT for Autoimmune Diseases: A Retrospective Study From the EBMT ADWP, IEWP, and PDWP Working Parties. Front Immunol (2019) 10:1570. doi: 10.3389/fimmu.2019.01570 PMC6622152 31333680

[9] Snowden JA Badoglio M Labopin M Giebel S McGrath E Marjanovic Z. Evolution, Trends, Outcomes, and Economics of Hematopoietic Stem Cell Transplantation in Severe Autoimmune Diseases. Blood Adv (2017) 1(27):2742–55. doi: 10.1182/bloodadvances.2017010041 PMC5745133 29296926

[10] Berman M Ben-Ami R Berliner S Anouk M Kaufman I Broyde A. The Effect of Tocilizumab on Inflammatory Markers in Patients Hospitalized With Serious Infections. Case Series and Review of Literature. Life (2021) 11(3):258. doi: 10.3390/life11030258 33804790 PMC8003879

[11] Admiraal R Lindemans CA van Kesteren C Bierings MB Versluijs AB Nierkens S. Excellent T-Cell Reconstitution and Survival Depend on Low ATG Exposure After Pediatric Cord Blood Transplantation. Blood (2016) 128(23):2734–41. doi: 10.1182/blood-2016-06-721936 27702800

[12] Admiraal R van Kesteren C Jol-van der Zijde CM Lankester AC Bierings MB Egberts TCG. Association Between Anti-Thymocyte Globulin Exposure and CD4+ Immune Reconstitution in Paediatric Haemopoietic Cell Transplantation: A Multicentre, Retrospective Pharmacodynamic Cohort Analysis. Lancet Haematol (2015) 2(5):e194–203. doi: 10.1016/S2352-3026(15)00045-9 26688094

[13] Knipp S Bier H Horneff G Specker C Schuster A Schroten H. Relapsing Polychondritis in Childhood – Case Report and Short Review. Rheumatol Int (2000) 19(6):231–4. doi: 10.1007/s002960000055 11063294

[14] Fonseca AR de Oliveira SK Rodrigues MC Aymoré IL Domingues RC Sztajnbok FR. Relapsing Polychondritis in Childhood: Three Case Reports, Comparison With Adulthood Disease and Literature Review. Rheumatol Int (2013) 33(7):1873–8. doi: 10.1007/s00296-011-2336-6 22210275

[15] Kingdon J Roscamp J Sangle S D’Cruz D. Relapsing Polychondritis: A Clinical Review for Rheumatologists. Rheumatology (2017) 57(9):1525–32. doi: 10.1093/rheumatology/kex406 29126262

[16] Moulis G Pugnet G Costedoat-Chalumeau N Mathian A Leroux G Boutémy J. Efficacy and Safety of Biologics in Relapsing Polychondritis: A French National Multicentre Study. Ann Rheum Dis (2018) 77(8):1172–8. doi: 10.1136/annrheumdis-2017-212705 29535124

[17] Rosen O Thiel A Massenkeil G Hiepe F Häupl T Radtke H. Autologous Stem-Cell Transplantation in Refractory Autoimmune Diseases After *In Vivo* Immunoablation and *Ex Vivo* Depletion of Mononuclear Cells. Arthritis Res Ther (2000) 2(4):327. doi: 10.1186/ar107 PMC17815 11056673

[18] Daikeler T Kötter I Bocelli Tyndall C Apperley J Attarbaschi A Guardiola P. Haematopoietic Stem Cell Transplantation for Vasculitis Including Behçet’s Disease and Polychondritis: A Retrospective Analysis of Patients Recorded in the European Bone Marrow Transplantation and European League Against Rheumatism Databases and a Review of the Literature. Ann Rheumatic Dis (2007) 66(2):202–7. doi: 10.1136/ard.2006.056630 PMC1798517 16950809

[19] Leroux G Costedoat-Chalumeau N Brihaye B Cohen-Bittan J Amoura Z Haroche J. Treatment of Relapsing Polychondritis With Rituximab: A Retrospective Study of Nine Patients. Arthritis Care Res (2009) 61(5):577–82. doi: 10.1002/art.24366 19405005

[20] Vitale A Sota J Rigante D Lopalco G Molinaro F Messina M. Relapsing Polychondritis: An Update on Pathogenesis, Clinical Features, Diagnostic Tools, and Therapeutic Perspectives. Curr Rheumatol Rep (2015) 18(1):3. doi: 10.1007/s11926-015-0549-5 26711694

[21] Zeuner M Straub RH Rauh G Albert ED Schölmerich J Lang B. Relapsing Polychondritis: Clinical and Immunogenetic Analysis of 62 Patients. J Rheumatol (1997) 24(1):96–101. 9002018

[22] Ouchi N Uzuki M Kamataki A Miura Y Sawai T. Cartilage Destruction Is Partly Induced by the Internal Proteolytic Enzymes and Apoptotic Phenomenon of Chondrocytes in Relapsing Polychondritis. J Rheumatol (2011) 38(4):730–7. doi: 10.3899/jrheum.101044 21239745

[23] Watanabe M Suzuki H Ara T Nishizuka M Morita M Sato C. Relapsing Polychondritis Complicated by Giant Cell Myocarditis and Myositis. Internal Med (2013) 52(12):1397–402. doi: 10.2169/internalmedicine.52.9080 23774555

[24] Buckner JH Van Landeghen M Kwok WW Tsarknaridis L. Identification of Type II Collagen Peptide 261–273-Specific T Cell Clones in a Patient With Relapsing Polychondritis. Arthritis Rheum (2002) 46(1):238–44. doi: 10.1002/1529-0131(200201)46:1<238::AID-ART10030>3.0.CO;2-M 11817597

[25] Hu FY Yan N Liang J Su R Gao C Xiao-Feng L. SAT0018 The Study of Cd4+t Cell Subsets In Recurrent Polychondritis. Ann Rheumatic Dis (2019) 78(Suppl 2):1074–. doi: 10.1136/annrheumdis-2019-eular.1250

[26] Rominger E BS Rose E Ferrada M Grayson P Colbert R Sikora K. T-Cell Receptor (TCR) Sequencing Reveals Decreased Diversity and Clonotypic Expansion of T-Cells in Relapsing Polychondritis (RP) [Abstract]. Arthritis Rheumatol (2020) 72 (suppl 10).

[27] Foidart J-M Abe S Martin GR Zizic TM Barnett EV Lawley TJ. Antibodies to Type II Collagen in Relapsing Polychondritis. New Engl J Med (1978) 299(22):1203–7. doi: 10.1056/NEJM197811302992202 714080

[28] Yang CL Brinckmann J Rui HF Vehring KH Lehmann H Kekow J. Autoantibodies to Cartilage Collagens in Relapsing Polychondritis. Arch Dermatol Res (1993) 285(5):245–9. doi: 10.1007/BF00371591 8379684

[29] Lutter L Spierings J van Rhijn-Brouwer FCC van Laar JM van Wijk F. Resetting the T Cell Compartment in Autoimmune Diseases With Autologous Hematopoietic Stem Cell Transplantation: An Update. Front Immunol (2018) 9(767). doi: 10.3389/fimmu.2018.00767 PMC5920130 29731752

[30] Delemarre EM van den Broek T Mijnheer G Meerding J Wehrens EJ Olek S. Autologous Stem Cell Transplantation Aids Autoimmune Patients by Functional Renewal and TCR Diversification of Regulatory T Cells. Blood (2016) 127(1):91–101. doi: 10.1182/blood-2015-06-649145 26480932

[31] Roord ST de Jager W Boon L Wulffraat N Martens A Prakken B. Autologous Bone Marrow Transplantation in Autoimmune Arthritis Restores Immune Homeostasis Through CD4+CD25+Foxp3+ Regulatory T Cells. Blood (2008) 111(10):5233–41. doi: 10.1182/blood-2007-12-128488 18256318

[32] Hoepfner S Haut PR O’Gorman M Kletzel M. Rapid Immune Reconstitution Following Autologous Hematopoietic Stem Cell Transplantation in Children: A Single Institution Experience. Bone Marrow Transplant (2003) 31(4):285–90. doi: 10.1038/sj.bmt.1703831 12621464

[33] Mackall CL Stein D Fleisher TA Brown MR Hakim FT Bare CV. Prolonged CD4 Depletion After Sequential Autologous Peripheral Blood Progenitor Cell Infusions in Children and Young Adults. Blood (2000) 96(2):754–62. doi: 10.1182/blood.V96.2.754.014k39_754_762 10887145

[34] Wiegering V Eyrich M Winkler B Schlegel PG. Comparison of Immune Reconstitution After Allogeneic Versus Autologous Stem Cell Transplantation in 182 Pediatric Recipients. J Pediatr Rheumatol Oncol (2019) 41(5):e302–e7. doi: 10.1097/MPH.0000000000001340 30418422

[35] Porrata LF Litzow MR Markovic SN. Immune Reconstitution After Autologous Hematopoietic Stem Cell Transplantation. Mayo Clinic Proc (2001) 76(4):407–12. doi: 10.1016/S0025-6196(11)62388-4 11322356

[36] Muraro PA Robins H Malhotra S Howell M Phippard D Desmarais C. T Cell Repertoire Following Autologous Stem Cell Transplantation for Multiple Sclerosis. J Clin Invest (2014) 124(3):1168–72. doi: 10.1172/JCI71691 PMC3934160 24531550

[37] Tsukamoto H Nagafuji K Horiuchi T Mitoma H Niiro H Arinobu Y. Analysis of Immune Reconstitution After Autologous CD34+ Stem/Progenitor Cell Transplantation for Systemic Sclerosis: Predominant Reconstitution of Th1 CD4+ T Cells. Rheumatology (2010) 50(5):944–52. doi: 10.1093/rheumatology/keq414 21172925

[38] Farge D Henegar C Carmagnat M Daneshpouy M Marjanovic Z Rabian C. Analysis of Immune Reconstitution After Autologous Bone Marrow Transplantation in Systemic Sclerosis. Arthritis Rheum (2005) 52(5):1555–63. doi: 10.1002/art.21036 15880600

[39] Dubinsky AN Burt RK Martin R Muraro PA. T-Cell Clones Persisting in the Circulation After Autologous Hematopoietic SCT Are Undetectable in the Peripheral CD34+ Selected Graft. Bone Marrow Transplant (2010) 45(2):325–31. doi: 10.1038/bmt.2009.139 19543329

[40] Muraro PA Douek DC Packer A Chung K Guenaga FJ Cassiani-Ingoni R. Thymic Output Generates a New and Diverse TCR Repertoire After Autologous Stem Cell Transplantation in Multiple Sclerosis Patients. J Exp Med (2005) 201(5):805–16. doi: 10.1084/jem.20041679 PMC2212822 15738052

[41] Alexander T Thiel A Rosen O Massenkeil G Sattler A Kohler S. Depletion of Autoreactive Immunologic Memory Followed by Autologous Hematopoietic Stem Cell Transplantation in Patients With Refractory SLE Induces Long-Term Remission Through *De Novo* Generation of a Juvenile and Tolerant Immune System. Blood (2009) 113(1):214–23. doi: 10.1182/blood-2008-07-168286 18824594

[42] Sun W Popat U Hutton G Zang YC Krance R Carrum G. Characteristics of T-Cell Receptor Repertoire and Myelin-Reactive T Cells Reconstituted From Autologous Haematopoietic Stem-Cell Grafts in Multiple Sclerosis. Brain (2004) 127(Pt 5):996–1008. doi: 10.1093/brain/awh117 14985264

[43] Zhang X Ye L Hu J Tang W Liu R Yang M. Acute Response of Peripheral Blood Cell to Autologous Hematopoietic Stem Cell Transplantation in Type 1 Diabetic Patient. PloS One (2012) 7(2):e31887. doi: 10.1371/journal.pone.0031887 22384093 PMC3285188

[44] Darlington PJ Touil T Doucet J-S Gaucher D Zeidan J Gauchat D. Diminished Th17 (Not Th1) Responses Underlie Multiple Sclerosis Disease Abrogation After Hematopoietic Stem Cell Transplantation. Ann Neurol (2013) 73(3):341–54. doi: 10.1002/ana.23784 23463494

[45] de Kleer I Vastert B Klein M Teklenburg G Arkesteijn G Yung GP. Autologous Stem Cell Transplantation for Autoimmunity Induces Immunologic Self-Tolerance by Reprogramming Autoreactive T Cells and Restoring the CD4+CD25+ Immune Regulatory Network. Blood (2006) 107(4):1696–702. doi: 10.1182/blood-2005-07-2800 16263787

[46] Zhang L Bertucci AM Ramsey-Goldman R Burt RK Datta SK. Regulatory T Cell (Treg) Subsets Return in Patients With Refractory Lupus Following Stem Cell Transplantation, and TGF-β-Producing CD8+ Treg Cells Are Associated With Immunological Remission of Lupus. J Immunol (2009) 183(10):6346–58. doi: 10.4049/jimmunol.0901773 PMC2784684 19841178

[47] Arruda LCM de Azevedo JTC de Oliveira GLV Scortegagna GT Rodrigues ES Palma PVB. Immunological Correlates of Favorable Long-Term Clinical Outcome in Multiple Sclerosis Patients After Autologous Hematopoietic Stem Cell Transplantation. Clin Immunol (2016) 169:47–57. doi: 10.1016/j.clim.2016.06.005 27318116

[48] Arruda LCM Clave E Moins-Teisserenc H Douay C Farge D Toubert A. Resetting the Immune Response After Autologous Hematopoietic Stem Cell Transplantation for Autoimmune Diseases. Curr Res Trans Med (2016) 64(2):107–13. doi: 10.1016/j.retram.2016.03.004 27316394

[49] Kong F-K Chen C-LH Cooper MD. Reversible Disruption of Thymic Function by Steroid Treatment. J Immunol (2002) 168(12):6500–5. doi: 10.4049/jimmunol.168.12.6500 12055271

[50] Fletcher AL Lowen TE Sakkal S Reiseger JJ Hammett MV Seach N. Ablation and Regeneration of Tolerance-Inducing Medullary Thymic Epithelial Cells After Cyclosporine, Cyclophosphamide, and Dexamethasone Treatment. J Immunol (2009) 183(2):823–31. doi: 10.4049/jimmunol.0900225 19564346

[51] Gaballa A Clave E Uhlin M Toubert A Arruda LCM. Evaluating Thymic Function After Human Hematopoietic Stem Cell Transplantation in the Personalized Medicine Era. Front Immunol (2020) 11(1341). doi: 10.3389/fimmu.2020.01341 PMC7412601 32849495

[52] Kinsella S Dudakov JA. When the Damage Is Done: Injury and Repair in Thymus Function. Front Immunol (2020) 11:1745. doi: 10.3389/fimmu.2020.01745 PMC7435010 32903477

[53] Marsh RA Lane A Mehta PA Neumeier L Jodele S Davies SM. Alemtuzumab Levels Impact Acute GVHD, Mixed Chimerism, and Lymphocyte Recovery Following Alemtuzumab, Fludarabine, and Melphalan RIC HCT. Blood (2016) 127(4):503–12. doi: 10.1182/blood-2015-07-659672 26644451

[54] Brinkman DMC de Kleer IM ten Cate R van Rossum MAJ Bekkering WP Fasth A. Autologous Stem Cell Transplantation in Children With Severe Progressive Systemic or Polyarticular Juvenile Idiopathic Arthritis: Long-Term Followup of a Prospective Clinical Trial. Arthritis Rheum (2007) 56(7):2410–21. doi: 10.1002/art.22656 17599770

[55] Snowden JA Badoglio M Alexander T. The Rise of Autologous HCT for Autoimmune Diseases: What Is Behind It and What Does It Mean for the Future of Treatment? An Update on Behalf of the EBMT Autoimmune Diseases Working Party. Expert Rev Clin Immunol (2019) 15(10):981–5. doi: 10.1080/1744666X.2019.1656526 31414932

[56] Gratwohl A. Allogeneic Hematopoietic Stem Cell Transplantation for Severe Autoimmune Diseases. Autoimmunity (2008) 41(8):673–8. doi: 10.1080/08916930802197677 18958760

[57] Snowden JA Kearney P Kearney A Cooley HM Grigg A Jacobs P. Long-Term Outcome of Autoimmune Disease Following Allogeneic Bone Marrow Transplantation. Arthritis Rheum (1998) 41(3):453–9. doi: 10.1002/1529-0131(199803)41:3<453::AID-ART11>3.0.CO;2-# 9506573

[58] Hinterberger W Hinterberger-Fischer M Marmont A. Clinically Demonstrable Anti-Autoimmunity Mediated by Allogeneic Immune Cells Favorably Affects Outcome After Stem Cell Transplantation in Human Autoimmune Diseases. Bone Marrow Transplant (2002) 30(11):753–9. doi: 10.1038/sj.bmt.1703686 12439698

[59] Bornhäuser M Kienast J Trenschel R Burchert A Hegenbart U Stadler M. Reduced-Intensity Conditioning Versus Standard Conditioning Before Allogeneic Haemopoietic Cell Transplantation in Patients With Acute Myeloid Leukaemia in First Complete Remission: A Prospective, Open-Label Randomised Phase 3 Trial. Lancet Oncol (2012) 13(10):1035–44. doi: 10.1016/S1470-2045(12)70349-2 22959335

[60] Bartelink IH Lalmohamed A van Reij EML Dvorak CC Savic RM Zwaveling J. Association of Busulfan Exposure With Survival and Toxicity After Haemopoietic Cell Transplantation in Children and Young Adults: A Multicentre, Retrospective Cohort Analysis. Lancet Haematol (2016) 3(11):e526–36. doi: 10.1016/S2352-3026(16)30114-4 PMC5159247 27746112

[61] Bacigalupo A Metafuni E Amato V Marquez Algaba E Pagano L. Reducing Infectious Complications After Allogeneic Stem Cell Transplant. Expert Rev Hematol (2020) 13(11):1235–51. doi: 10.1080/17474086.2020.1831382 32996342

[62] Sullivan KM Sarantopoulos S. Allogeneic HSCT for Autoimmune Disease: A Shared Decision. Nat Rev Rheumatol (2019) 15(12):701–2. doi: 10.1038/s41584-019-0306-7 31530942

